# 60 years ago, Francis Crick changed the logic of biology

**DOI:** 10.1371/journal.pbio.2003243

**Published:** 2017-09-18

**Authors:** Matthew Cobb

**Affiliations:** School of Biological Sciences, University of Manchester, Manchester, United Kingdom

## Abstract

In September 1957, Francis Crick gave a lecture in which he outlined key ideas about gene function, in particular what he called the central dogma. These ideas still frame how we understand life. This essay explores the concepts he developed in this influential lecture, including his prediction that we would study evolution by comparing sequences.

## Introduction

This month marks the 60th anniversary of one of the most significant lectures in the history of biology. It was given on 19 September 1957 by Francis Crick as part of a Society for Experimental Biology symposium on the Biological Replication of Macromolecules, held at University College London. Originally entitled ‘Protein synthesis,’ the title acquired a magisterial introductory ‘On’ during writing up for publication the following year [1]. The lecture went far further than its title suggested: as Crick pointed out in the opening paragraph, he also addressed ‘the other central problems of molecular biology—those of gene action and nucleic acid synthesis.’

Crick’s talk is now often called the ‘central dogma’ lecture, for it was here that he first publicly presented this frequently misunderstood concept. While this was highly significant, the content of the lecture was even richer—it also saw Crick outline his view of the nature of life and of genetic information and the source of protein folding as well as making two bold and spectacularly accurate predictions: that there must exist a small ‘adaptor’ molecule (now known as tRNA) that could bring amino acids to the site of protein synthesis and that in the future, scientists would be able to explore rich evolutionary sources of information by comparing sequence data. In this one brief lecture, Crick profoundly influenced how we think. In *The Eighth Day of Creation*, journalist Horace Judson went so far as to claim that on that day 60 years ago, Crick “permanently altered the logic of biology [2].”

### Crick’s presentation

Crick’s hour-long lecture was given on the third day of a leisurely 4-day meeting (at most four talks a day), with participants from France, the United States, Belgium, and Hungary as well as a solid contingent of Britons. One of the French speakers was molecular geneticist François Jacob, for whom this was his first encounter with Crick. The impression Crick made was lasting—30 years later, Jacob recalled the lecture:

“Tall, florid, with long sideburns, Crick looked like the Englishman seen in illustrations to 19th century books about Phileas Fogg or the English opium eater. He talked incessantly. With evident pleasure and volubly, as if he was afraid he would not have enough time to get everything out. Going over his demonstration again to be sure it was understood. Breaking up his sentences with loud laughter. Setting off again with renewed vigour at a speed I often had trouble keeping up with…Crick was dazzling.” [3]

There is no manuscript of Crick’s actual talk, only the 11,000-word article that was published in 1958, which Crick prepared for publication in October 1957. [4] This version would presumably have been too long for Crick to read out in his 60-minute slot, even if he did speak incredibly quickly and, as he recalled, ‘ran overtime’ [2]. According to the acknowledgement in the paper, the version with which we are all familiar was the product of many discussions with Sydney Brenner, who also played a role in “redrafting” the manuscript, presumably for publication.

Crick’s opening statement may seem unsettling to the modern reader:

“I shall…argue that the main function of the genetic material is to control (not necessarily directly) the synthesis of proteins. There is a little direct evidence to support this, but to my mind the psychological drive behind this hypothesis is at the moment independent of such evidence.”

This highlights how uncertain scientists were at the time about gene function—as Crick pointed out, at the time, not everyone accepted that nucleic acids were involved in protein synthesis [5]. In 1957, ribosomes were known only as microsomes, and their function and composition was uncertain; messenger RNA was still undreamt of—it would be properly identified only in the summer of 1960, and the discovery was not published until the following year [6,7,8].

Faced with the lack of experimental evidence as to how genes produced proteins, Crick fell back on what he excelled in: outlining general, bold concepts that drew together a wide variety of strands into a compelling whole. As he recalled: “In looking back I am struck…by the brashness which allowed us to venture powerful statements of a very general nature [9].”

### Protein synthesis and the sequence hypothesis

Crick had been thinking at a very high level about the relation between DNA, RNA, and protein for several years, partly inspired by documents and letters that were exchanged between members of the 20-strong RNA Tie Club, a loose discussion group that included Brenner, Jim Watson, and a host of physicists and mathematicians, led by George Gamow [10]. In 1954, Watson wrote a series of letters to Crick as he tried to grapple with the role of RNA, which he jokingly called ‘the mysteries of life’ [11]. Watson initially thought that DNA might be chemically converted into RNA but gradually shifted his view and ended up arguing that DNA acted as a template for RNA, an answer he described as ‘not ugly’ [12].

Crick took these ideas and the experimental data that increasingly suggested that RNA was some kind of intermediate between DNA and protein (these data referred to ribosomes rather than mRNA) and developed a scheme to explain the relations between these three classes of biological molecules. In so doing, he had to get to grips with what exactly was in a gene and what took place if DNA was used as a template for RNA—not in biochemical terms, but in the most abstract way possible.

To do this, Crick had to resolve an issue that had been perplexing scientists since he and Watson introduced the concept of “genetic information” in their second, less often-read 1953 *Nature* article [13]. Although the idea had been rapidly and widely adopted, no one was clear what exactly genetic information might consist of. In his 1957 lecture, Crick gave a disarmingly straightforward definition—information in this context was simply ‘the determination of a sequence of units.’ This highlighted the existence of a link between the base sequences of nucleic acids and those of amino acids in a protein—they pointed to the reality of the genetic code. This in turn enabled Crick to conceptualize the link between gene and protein. He called this link “the flow of information” and added this concept to the factors that were generally accepted to describe protein synthesis and, indeed, life itself—the flow of matter and the flow of energy.

This definition of information raised a problem. Proteins are 3-dimensional (3D) structures whereas a DNA sequence is 1-dimensional (1D). Crick recognized that there might be some unknown source of information that enabled proteins to fold, but he argued that the ‘more likely hypothesis’ was that ‘folding is simply a function of the order of the amino acids.’ In other words, 3D protein structure is an emergent property of the 1D sequence. This simple ‘sequence hypothesis,’ as he termed it, remains essentially true today, despite the acknowledged role of molecular chaperones.

### The central dogma

The most widely known of the powerful statements made by Crick in his lecture related to the flow of information between genes and proteins [14]. He had been musing about this for some time and in October 1956 wrote a set of notes entitled ‘Ideas on protein synthesis’ that took up 2 pages [15]. The second sentence of this document read, “The Central Dogma: ‘Once information has got into a protein it can’t get out again. Information here means the sequence of the amino acid residues, or other sequences related to it.’” This statement was repeated several times in the September 1957 lecture and also appeared in a *Scientific American* article on nucleic acids, which Crick published in October 1957 [16].

**Fig 1:**
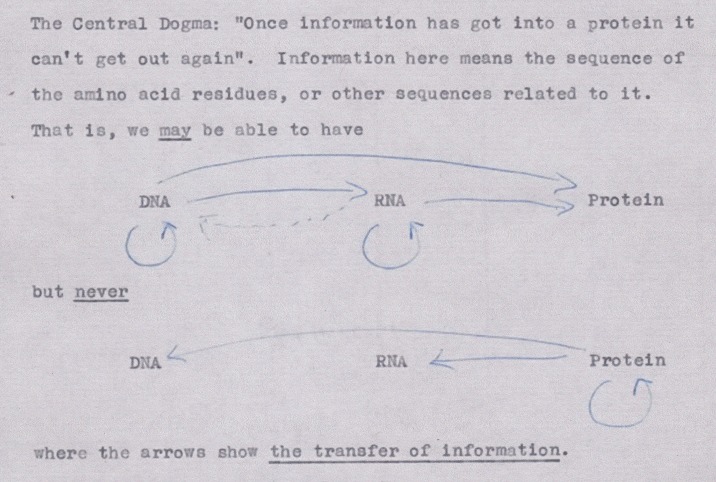
Crick’s first outline of the central dogma, from an unpublished note made in 1956. Credit: Wellcome Library, London.

**Fig 2:**
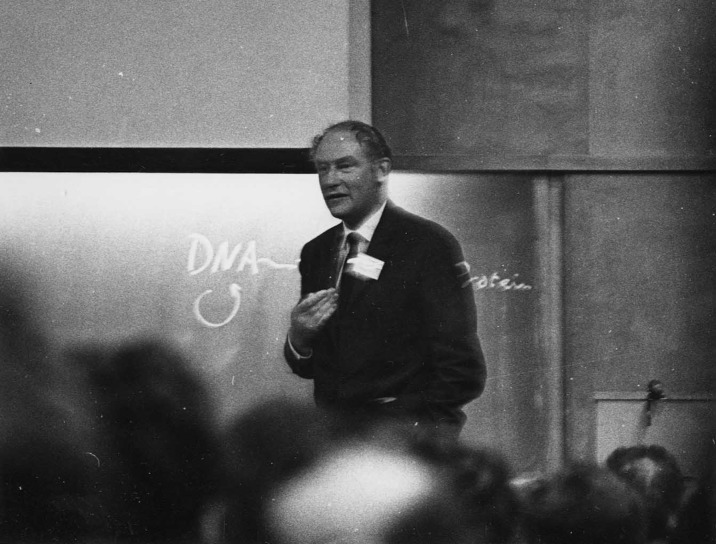
Crick speaking at the 1963 Cold Spring Harbor Symposium. Note the drawing of the central dogma on the blackboard. *Credit*: *Cold Spring Harbor Laboratory*.

In Crick’s 1956 notes, this definition of the central dogma was followed by a diagram illustrating his idea, with arrows drawn in blue biro (Fig 1). This figure was never published, although Crick did draw it on the blackboard when giving talks (see Fig 2, from 1963—he may have done something similar in September 1957), and a slightly amended version was eventually published in 1970 [17].

For Crick, four kinds of information transfer clearly existed: DNA → DNA (DNA replication), DNA → RNA (the first step of protein synthesis), RNA → protein (the second step of protein synthesis) and RNA → RNA (RNA viruses copying themselves). There were two steps for which there was no evidence but that Crick thought were possible (hence the dotted lines in the figure): DNA → protein (this would mean RNA was not involved in protein synthesis) and RNA → DNA (structurally possible, but at the time, there no was no perceptible biological function).

Just as striking were the three flows of information that Crick considered to be impossible due to both lack of evidence and lack of biochemical mechanism. These were protein → protein, protein → RNA, and above all, protein → DNA. This was what Crick meant when he said that once information had gone from DNA into the protein, it could not get out of the protein and go back into the genetic code. This is the central dogma.

Crick admitted that the direct evidence for this hypothesis was ‘negligible’ and that it had a ‘speculative nature,’ but he defended his approach by pointing out that cosmologists had no qualms about constructing theories without adequate experimental data. That implicit comparison with grand theories of the universe is justified, for Crick was laying out the foundations of a new way of understanding how the cell works. The simplicity of the sequence hypothesis and the central dogma, together with the focus on information, brought a clear explanatory power to the synthesis of protein molecules that could take virtually any form and could ‘do almost anything,’ as Crick put it. Once the cell’s fundamental activity was conceived of in this way, everything fell into place. Crick advised his listeners to attempt to explain protein synthesis without these two basic principles—it was ‘an instructive exercise,’ he said. ‘One generally ends in the wilderness,’ he claimed.

Students are now often mistakenly taught that the central dogma is something like ‘DNA → RNA → protein’ (as popularised by Watson in his 1965 textbook *Molecular Biology of the Gene* [18]) or, even less precisely, ‘DNA makes RNA makes protein’ (as first suggested by Jean Brachet in 1960 [19]). This view, which went back to André Boivin in 1949 [20] and Alexander Dounce in 1953 [21], was very different to what Crick had in mind (it also confuses students, who often fail to grasp what the arrows mean or ‘makes’ implies [22]).

In 1970, following the discovery by Howard Temin and David Baltimore of reverse transcriptase, which enables information to flow in the direction RNA → DNA, *Nature* published an editorial entitled ‘Central dogma reversed’ [23]. Crick wrote a slightly tetchy response, repeating what he had actually stated in 1957, and rightly insisting that he had never argued that RNA → DNA was impossible [17]. In a distinctly undogmatic approach, he emphasised that our knowledge of cell biology was remarkably limited and that surprises might be in store, pointing to the example of the disease scrapie in which a protein seemed to act as an infectious agent (Stanley Prusiner later described this as a prion). However, even in the case of scrapie and other prion diseases, infection involves a change in conformation, not de novo synthesis.

Crick’s essential argument still holds: protein synthesis relies on nucleic acids, and once the genetic information has got into the protein, it cannot alter the DNA sequence. Despite recent excitement about transgenerational epigenetic inheritance due to histone modifications, DNA methylation, or other temporary modifications of material surrounding the genetic sequence, there is no evidence in any organism that the information in a DNA sequence can be rewritten from information in a protein.

In one aspect of the central dogma, Crick was mistaken. In reality, the ‘Central Dogma’ was anything but a dogma. Crick later claimed that he had not properly understood the meaning of ‘dogma’—Jacques Monod had to explain to him exactly what it meant. An indication of the truth of this assertion can be seen in the lecture when he states that the name that he has coined emphasizes the speculative nature of the idea—a dogma is not speculative. As Crick later acknowledged, a more accurate description would have been ‘basic assumption’ [17]. This does not sound quite so sexy, but it would have removed a lot of subsequent misunderstanding. Perhaps if Crick had not used such a dramatic turn of phrase, many subsequent critics would not have become so exercised about the question.

### RNA and the adaptor

Crick used his lecture to publicly air another key idea about protein synthesis that he had been developing in private. In 1955, he circulated a note to the RNA Tie Club entitled ‘On degenerate templates and the adaptor hypothesis’ [24]. In this document, he argued that it was structurally impossible for any nucleic acid to act as a template for a particular amino acid; the duo of Crick and Brenner therefore came up with what Brenner called ‘the adaptor hypothesis’—an unknown class of molecule that would act like an electric plug adaptor, taking amino acids to the ribosome for protein assembly.

Crick was understandably unable to predict the nature of these adaptor molecules, but he felt that it was likely that they would contain nucleotides, which would be able to pair with both DNA and the RNA site of protein synthesis. Even allowing for the fact that he did not yet fully grasp the role of ribosomal RNA, Crick’s vision was astonishingly clear:

“The template could consist of perhaps a single chain of RNA…Each adaptor molecule containing, say, a di- or trinucleotide would each be joined to its own amino acid by a special enzyme. These molecules would then diffuse to the microsomal particles and attach to the proper place on the basis of the RNA by base-pairing.”

Crick and Brenner’s prediction would soon be proven correct—as Crick was giving his talk, Hoagland and Zamecnik were putting the finishing touches to their paper describing the isolation of the adaptor, which was eventually called tRNA [25].

### Crick and evolutionary biology

There were two aspects of Crick’s lecture that related to evolutionary thinking. The first was that the central dogma supported the neo-Darwinian view that it was impossible for any character that was acquired during an organism’s life to affect its hereditary characters. This provided support for the widespread hostility to the view that had been held by Darwin, Lamarck, and others, according to which, patterns of use and disuse could lead to changes in the frequency of characters in subsequent generations.

Although in most organisms, including bacteria, plants, and even some animals, there is no separation between the copies of DNA used for protein synthesis and those used for transmitting genetic information to the next generation, Crick could see no conceivable mechanism whereby changes acquired during life could feed back into the DNA sequence. This was later considered to be an additional argument against Lamarckian inheritance and a reinforcement of Weismann’s separation of the germ and somatic cell lines (something that applies only to most animals) [2]. However, Crick did not mention either of these ideas.

The other evolutionary aspect to Crick’s lecture came in a brief and little-noticed aside, in which he essentially predicted the development of phylogenetics. In 1957, protein sequencing was extremely primitive, while sequencing DNA was two decades in the future. Complete amino acid sequences for insulin had been described for just five species, but nevertheless, Crick could see the way things would go. In an incredibly prescient prediction, he stated:

“Biologists should realise that before long we shall have a subject which might be called ‘protein taxonomy’—the study of the amino acid sequences of the proteins of an organism and the comparison of them between species. It can be argued that these sequences are the most delicate expression possible of the phenotype of an organism and that vast amounts of evolutionary information may be hidden away within them.”

This insight appears to have had little impact on thinking about the potential power of studying sequences—the history of bioinformatics [26] is generally traced back to the work of Dick Eck [27], Margaret Dayhoff [28], and Emile Zuckerkandl and Linus Pauling [29] in the early 1960s, none of whom cited Crick’s lecture. Further exploration of the work of the early bioinformaticians may reveal currently-unknown direct connections with Crick’s ideas, but whatever the case, the clarity of this vision underlines the power of Crick’s thinking.

## Conclusion

It took some time for Crick’s lecture to exert its influence. Despite Jacob’s vivid description of how Crick presented his ideas, there is no indication that the content immediately changed the thinking of those in the audience. Only one of the other presentations at the symposium made any reference to Crick’s novel ideas in the revised printed version, and even here, the authors appear to have thought that Crick was indeed being dogmatic in his views because he speculated rather than strictly limiting himself to the experimental evidence [30].

Since then, the renown of the lecture has grown, and it has been cited over 800 times. The pattern of citations is U-shaped, with an early peak of 28 in 1962, followed by a trough of a handful of citations per year between 1971 and 1990, rising to 52 citations in 2014. Crick was later quite harsh on his lecture, describing it as ‘a mixture of good and bad ideas, of insight and nonsense’ [9]. This seems unfair—any nonsense is primarily due to lack of experimental evidence at the time. The reason why people still return to a 60-year-old lecture is because of the power of its ideas and the clarity with which they are presented. Crick’s style and intellectual verve continue to be both influential and inspirational; everyone should read or reread this brilliant lecture by one of the 20th century’s greatest scientists, a lecture that changed how we think.
